# Looking the Cow in the Eye: Deletion in the *NID1* Gene Is Associated with Recessive Inherited Cataract in Romagnola Cattle

**DOI:** 10.1371/journal.pone.0110628

**Published:** 2014-10-27

**Authors:** Leonardo Murgiano, Vidhya Jagannathan, Valerio Calderoni, Monika Joechler, Arcangelo Gentile, Cord Drögemüller

**Affiliations:** 1 Institute of Genetics, Vetsuisse Faculty, University of Bern, Bern, Switzerland; 2 Bovine practitioner, Fontanelice, Italy; 3 Department of Veterinary Medical Sciences, University of Bologna, Ozzano dell'Emilia, Italy; Innsbruck Medical University, Austria

## Abstract

Cataract is a known condition leading to opacification of the eye lens causing partial or total blindness. Mutations are known to cause autosomal dominant or recessive inherited forms of cataracts in humans, mice, rats, guinea pigs and dogs. The use of large-sized animal models instead of those using mice for the study of this condition has been discussed due to the small size of rodent lenses. Four juvenile-onset cases of bilateral incomplete immature nuclear cataract were recently observed in Romagnola cattle. Pedigree analysis suggested a monogenic autosomal recessive inheritance. In addition to the cataract, one of the cases displayed abnormal head movements. Genome-wide association and homozygosity mapping and subsequent whole genome sequencing of a single case identified two perfectly associated sequence variants in a critical interval of 7.2 Mb on cattle chromosome 28: a missense point mutation located in an uncharacterized locus and an 855 bp deletion across the exon 19/intron 19 border of the bovine nidogen 1 (*NID1*) gene (c.3579_3604+829del). RT-PCR showed that *NID1* is expressed in bovine lenses while the transcript of the second locus was absent. The *NID1* deletion leads to the skipping of exon 19 during transcription and is therefore predicted to cause a frameshift and premature stop codon (p.1164fs27X). The truncated protein lacks a C-terminal domain essential for binding with matrix assembly complexes. *Nidogen 1* deficient mice show neurological abnormalities and highly irregular crystal lens alterations. This study adds *NID1* to the list of candidate genes for inherited cataract in humans and is the first report of a naturally occurring mutation leading to non-syndromic catarct in cattle provides a potential large animal model for human cataract.

## Introduction

Cataract is a known condition affecting the internal integrity of the eye lens (crystalline) and leads to its opacification, interfering with normal sight. Vision can be compromised up to partial or total blindness, especially if both eyes are affected (bilateral cataract) [1]–[3]. The etiology of cataracts is heterogeneous like trauma, radiation, chronic disease, drugs or medications [4], [5]. A very important triggering factor of cataracts is aging; the majority of cataract cases manifest themselves in the later stages of life [6]. It is thought that nutrition could play a role in cataract development as well [7]. Nonetheless, a number of cases of cataracts are congenital or have an early onset [8], [9]. Approximately 1/3 of all congenital cataracts are caused by mostly autosomal dominant, rarely autosomal recessive or X-linked inherited mutations [9]–[12]. Almost 200 genes and a great number of loci identified as being causative of Mendelian and age-related cataract in humans are known [13]. The affected genes encode various proteins of importance for the crystalline structural stability [2], [12] most of them being crystallins, connexins and membrane junction proteins, but transcription factors like MAF are present as well [2], [13]. Many causative mutations change protein conformation and decrease their solubility and stability [12], [14] very likely leading to self-aggregation and precipitation [15].

In animals, most of the knowledge regarding cataractogenesis comes from genetic studies of hereditary cataracts in mice. Mutations causing inherited non-syndromic cataract have been identified in at least 18 mouse genes [16], and the use of animal models for the condition has been discussed for long time [17]. However, the need for a medium sized animal model has been pointed out due to the fact that rodents have small lenses which are hard to dissect and manipulate and need to be pooled for analysis. [18]. As an example, dogs showing breed specific forms of inherited cataract have been proposed as a complementary large animal model for molecular studies regarding cataract development, with the purpose of facilitating gene discovery and the development of therapies (OMIA-9615, [16]).

The occurrence of cataract in livestock probably is underestimated. This is somehow expected since, compared to other condition of greater severity, cataracts does not affect the health of the animal in such a way to hamper its production efficiency. One study has mapped a locus for an inherited cataract form in sheep to a region on chromosome 6 [19]. The authors stressed the necessity of developing a genetic test for cataract in sheep as predicting which animals will develop cataracts would make them suitable models for the development of the condition and to study possible therapies [19]. Indeed, the use of a sheep model for cataracts in which the potential therapeutic efficacy of a chemical has been tested has been reported [20]. Cataracts in cattle have been reported sporadically (OMIA-9913) and the mode of inheritance has been an object of debate [21], [22]. A recent case of congenital cataract in Ayrshire cattle with a high prevalence in a single herd was reported, but a genetic etiology has not been demonstrated [23], as well as in a population of Swiss calves [24]. One study has reported a dominant mutation in the *FNB1* gene as the cause of Marfan syndrome in cattle accompanied by cataract beside other defects [25].

Romagnola is a local variety of beef cattle bred in north central Italy of about twenty thousand animals. Due to a significant level of inbreeding, the Romagnola breed recently has experienced outbreaks of two recessive diseases, namely paunch calf syndrome and pseudomyotonia, for which gene tests for eradication were developed after successful identification of the causative gene mutations [26]–[27]. During the fall of 2013 cataracts in four inbred juvenile Romagnola cattle were observed, prompting an in depth study to identify the causative mutation.

## Results and Discussion

### A recessively inherited juvenile-onset form of cataract in cattle

**Figure 1:**
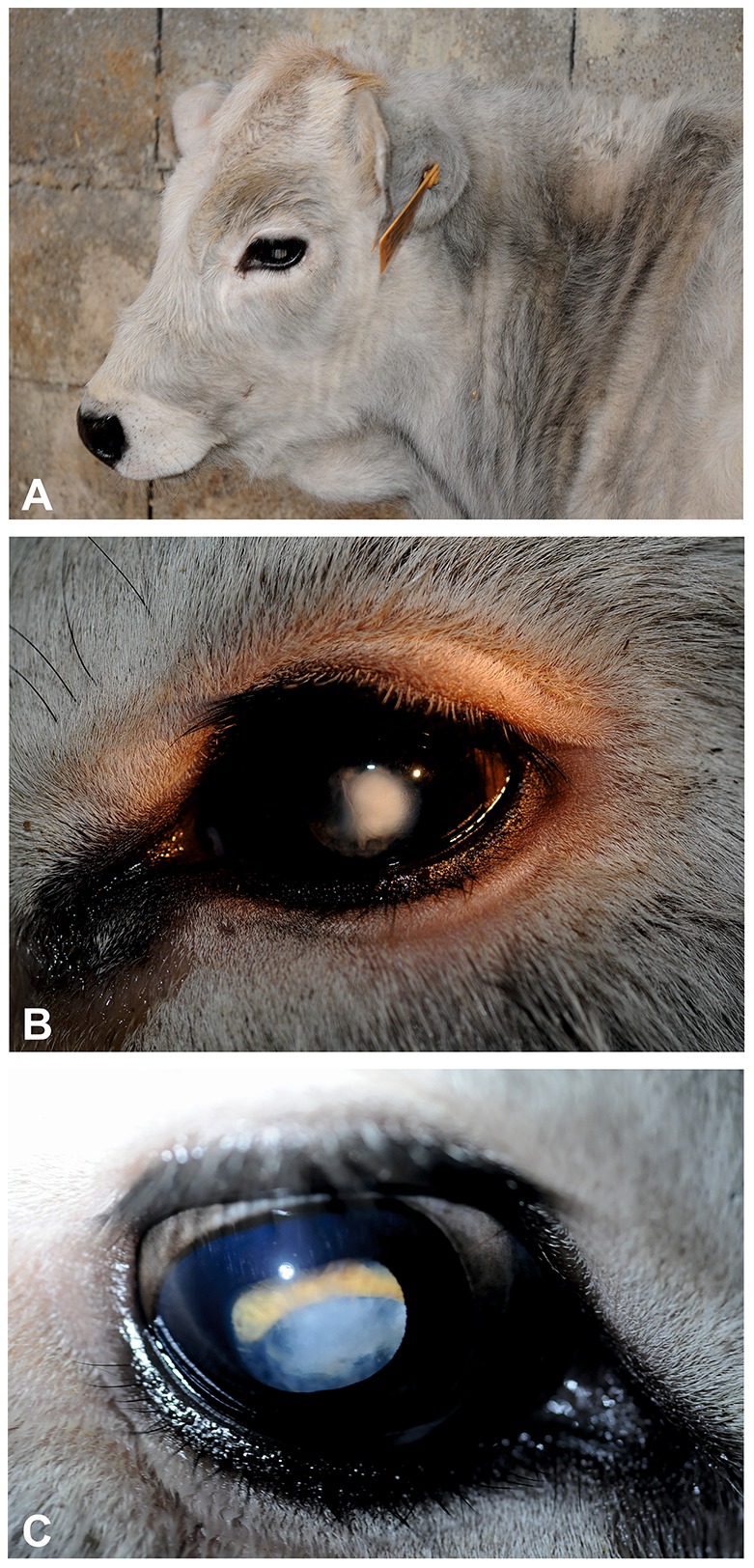
Cataract in Romagnola cattle. (**A**) Opacity of the left eye's noted from a distance. Case 3, born 27 July 2013, photo 4^th^ November 2013. (**B**) Dioptric media opacity of the left eye. Case 2, born 16 ^th^ August 2013, photo 6 ^th^ November 2013. (**C**) Mild central nuclear opacity. Fundus only partially to view (yellow  =  tapetal retina; blue  =  papilla). Case 2, born 16 ^th^ August 2013, photo 6 ^th^ November 2013.

In November 2013, four male Romagnola calves, varying in age from 3 to 4 months, were seen due to a visible clouding of both eyes causing obvious signs of impaired vision (**Figure 1**). The problem had been noticed by the owner some weeks prior to the request for consultation. According to the records of the owner the opacity was not present at birth. The clinical investigation carried out in dim light during the first on-farm visit showed a relatively less severe opacity of the lenses. Vision impairment was evident when the animal moved within the box, especially when forced to escape rapidly from the examiner. The body condition score of the animals was slightly reduced, whereas mental status was normal. According to the owner, all dams of the affected calves, as well as the other animals present at the farm, had no visible ocular problems. In addition, one of the affected animals also showed a slight head tilt towards the right side (**Video S1**). The cow-calf herd is made up of 60 Romagnola cows housed in a free stall barn with straw as bedding material, and are fed hay and barley-based concentrate (80% barley +20% mais). In the summer (May to November), the cows are allowed to graze in a hilly pasture and in a wood near the farm, which is also populated with wild animals. The hay and the concentrate are supplemented at the end of the grazing period when grass is reduced due to the dry season. The calving season is concentrated in winter and spring (November to May). Two sires are present in the herd and they are usually kept for a maximum of three years to avoid parental inbreeding. Cows and heifers are vaccinated annually against bovine viral diarrhea (BVD), rhinotracheitis (IBR), parainfluenza 3 (PI3), and bovine respiratory syncitial virus (BRSV) viruses using inactivated strains. At the time of our farm visit, the herd had not been treated against parasites for long time, and, in fact, gastrointestinal strongyles were detected using coprological investigation. During the first visit no elements suggestive of nutritional or potentially toxic environmental factors were detected. The suggested correlation of the cataracts to the proximity of mobile telephone masts [24] was excluded on the basis of the absence of such stations in the surrounding area.

One calf was admitted to the teaching hospital of the Department of Veterinary Medical Sciences of the University of Bologna for in-depth clinical study. A thorough ophthalmologic examination was carried out in a darkened environment. The exam did not show any lid or and conjunctival problems, the menace was bilaterally positive, and the Shirmer tear tests as well as the intraocular pressure (IOP) values were normal. By direct focal light source stimulation, the direct and consensual pupillary reflexes were normal or slightly more accentuated. Direct ophthalomoscopy revealed a regular morphology and arrangement of the iris and net margins of the pupillary lumen. After mydriasis induction, examination of both lenses revealed mild central nuclear opacity irregularly extending towards the periphery. Partial fundus evaluation revealed a regular papilla and a normal tapetum. Hematological parameters and clinical biochemistry did not show notable alterations. The final diagnosis for this calf was bilateral incomplete immature nuclear cataract.

**Figure 2:**
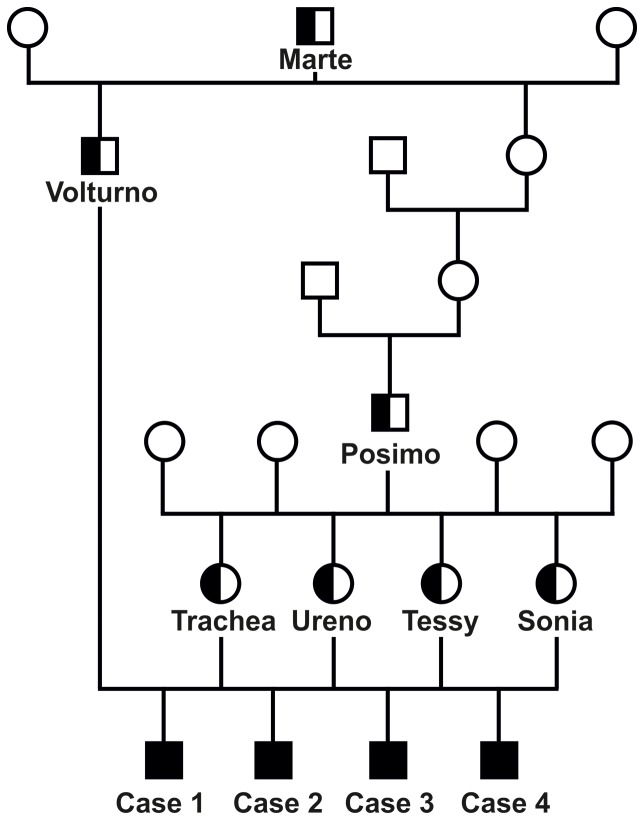
Family tree showing four Romagnola calves affected by cataract and their parents. Males are represented by squares, females by circles. Affected animals are shown with fully black symbols and genotyped carriers with a half-filled symbol. All unnamed animals with empty symbols were not available for genotyping. Note the inbreeding loop to the sire *Marte*.

Analysis of the pedigree data revealed that all four affected animals were paternal half siblings of *Volturno* (**Figure 2**). Furthermore, their respective dams were also paternal half sibs belonging to another sire *Posimo* which had been culled some years before. We examined three other young calves with the same family relationship and approximately the same age as the affected animals and they did not show any visible ocular problems. Both bulls, *Volturno* and *Posimo*, had a common male ancestor (*Marte*) over 1 and 3 generations ago which was born in 1978. Although none of these three sires was still alive a visible cataract could probably be excluded as they were selected after a detailed clinical exam revealing no signs of genetic conditions before they were used as artificial insemination sires. Taken together, we assumed a monogenic recessive inheritance of the underlying mutation causing the cataract phenotype in that family of Romagnola cattle.

### Mapping of the cataract mutation to a 7.2 Mb region on BTA 28

**Figure 3:**
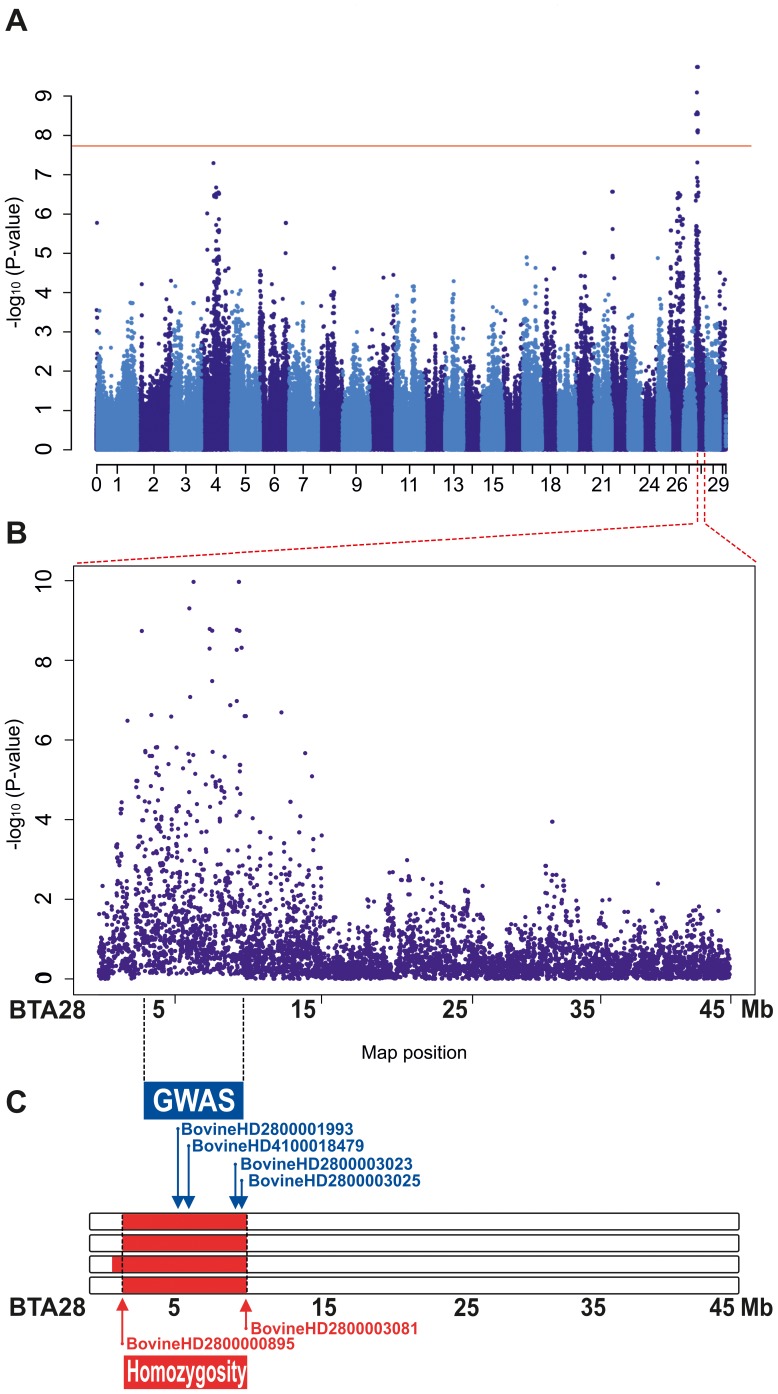
Mapping of the cataract mutation to BTA 28. (**A**) Results of the genome-wide association study (GWAS): Manhattan plot showing the negative log of the raw P values calculated with the mixed-model genotypic association test. Genome-wide significance thresholds are indicated (P≤0.01, solid red horizontal line at 7.7 10^−8^ –log_10_Pvalue). (**B**) Detailed view of Manhattan plot for chromosome 28. (**C**) (blue) the 4 most significantly associated BTA 28 SNPs from GWAS in combination with the identical by descent (IBD; red) segments of the four affected animals. The names of the SNPs lines are reported along with their positions on the map.

Hypothesizing a simple Mendelian recessive inheritance we initiated a positional cloning study to unravel the underlying genetics. Initially we genotyped 777,961 evenly spaced SNPs on the 4 affected calves, the sire and the 4 dams, and we merged these data along with a set of similar genotype data of 51 Romagnola cattle which had been generated in the course of a previous study [26]. After removing 236,126 non-informative markers, 549,341 SNPs were used for genome-wide association mapping (GWAS). The calculated genomic inflation factor (lambda) was 1.78 suggesting a fairly highly stratified population. We therefore decided to carry out the analysis using a mixed-model in order to take into account the population stratification; this showed significantly associated markers exceeding the threshold for genome-wide significance after a Bonferroni correction (raw P value <7.7×10^−8^). A best 14 markers were located in the same single contiguous genomic region (3.32–10.05 Mb) on bovine chromosome 28 (BTA 28) (**Figure 3A** **+B**; **Figure S1**; **Table S1**).

In the light of the highly likely monogenic recessive inheritance (**Figure 2**) we assumed that the four affected animals were expected to be identical by descent (IBD) for the causative mutation and flanking chromosomal segments. Therefore, we searched for extended regions of homozygosity with simultaneous allele sharing and found five genome regions greater than 1 Mb that fulfilled these criteria whereas the largest homozygous segment corresponds to a 7.2 Mb interval from 2,870,897 to 10,073,583 on BTA 28 (**Figure 3C**; **Table S2**). Observing an almost matching overlap of the GWAS hit and the homozygous region on chromosome 28 we concluded that this single genome interval probably contains the causative mutation for the cataract phenotype. The mapped critical interval contains 42 annotated genes beside a number of uncharacterized loci. Consulting the database to understand their expected function revealed that the region contained no obvious candidates, except possibly tubulin folding cofactor E (*TBCE*) since tubulin folding is supposed to have a role in lens transparency [28].

### A deletion in the *NID1* gene is associated with cataract in cattle

**Figure 4:**
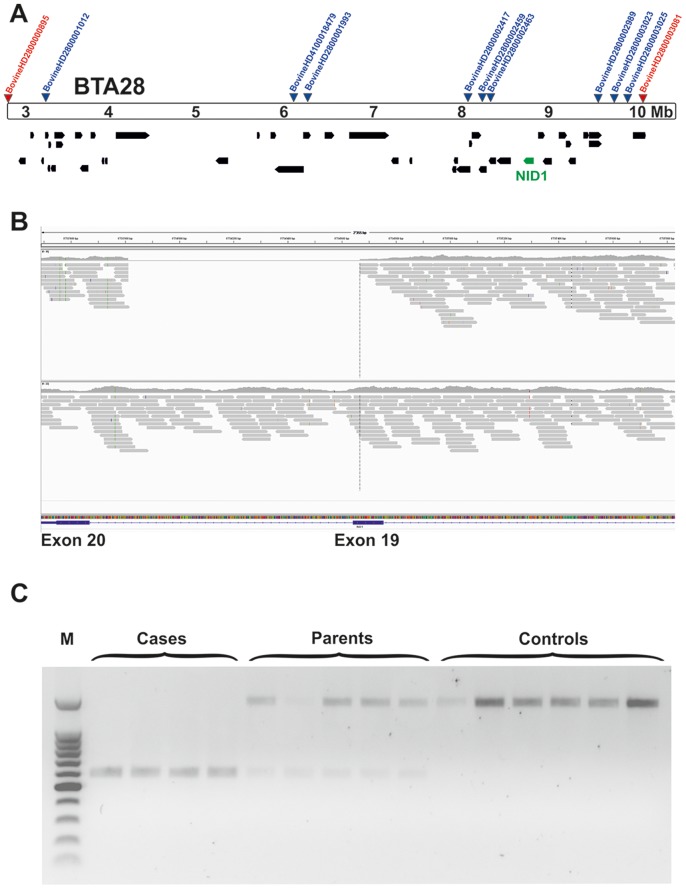
The *NID1* deletion. (**A**) Gene content of the fine-mapped critical interval Position of the two extreme homozygous marker (red) from homozygosity mapping and of the nine most significant markers from GWAS (blue) are indicated. Annotated genes in the region are shown in black, along with *NID1* (green). (**B**) Screenshot of the next generation sequence reads mapped against the reference sequence. Note the 855 bp deletion. (**C**) Detected genotypes of the *NID1* deletion. Note the shorter PCR product in affected animals, the longer in controls and the presence of both products in carriers.

For mutation analysis we sequenced the entire genome of a single affected animal (case 2, **Figure 2**) in order to detect all the variants in the annotated genes and loci of the mapped 7.2 Mb interval on BTA 28. We collected 319,462,911 100 bp paired-end reads from a shotgun fragment library corresponding to roughly 13.5× coverage of the genome. The SNPs and short indel variants were called compared to the reference genome and 92,955 high quality variants across the whole exome including untranslated regions and 10 bp of flanking introns were detected, of which 27,597 were coding variants. Within the exome of the critical BTA 28 region we detected a total of 129 variants, of which 43 were located within coding sequences or within splice sites having a predicted effect on the amino acid sequence of the annotated genes (**Table S3**). Comparison of the DNA variants between the affected calf and 44 cow genomes of 15 different cattle breeds that had been sequenced in our laboratory in the course of other studies revealed that 42 of the coding variants occurred in the control genomes of animals from different breeds and could thus be excluded as causative variants. The single remaining variant was a missense mutation in the uncharacterized locus *ENSBTAG00000039845* (c.687G>C, p.Q229H). Furthermore, we used the Delly package [29] for looking for larger deletions in the sequenced case and in 41 control cow genomes with a genome-wide coverage of more than 10 fold. A total of 253 deletions were private structural variants occurring only in the genome of the affected Romagnola animal with cataract of which a single 855 bp deletion was detected in the critical region on BTA 28 (g.8733811_8734665del). Annotation showed that this deletion removes 26 nucleotides from the 3′-end of exon 19 (nts 3579 to 3604) and 829 nucleotides from the 5′-end of intron 19 (nts 3604+1 to +827) of of the bovine nidogen 1 (*NID1*) gene (c.3579_3604+829del; **Figure 4A** **+B**). We genotyped the two detected private coding variants in larger cohorts of Romagnola cattle. The *NID1* deletion was validated by PCR and subsequent agarose gel electrophoresis confirmed that all four affected animals were homozygous, while all five parents were heterozygous, which was confirmed by subsequent Sanger sequencing (**Figure 4C**). Later on, fragment size analyses were performed for *NID1* genotyping. PCR and Sanger sequencing was used for validation and genotyping of the *ENSBTAG00000039845* SNP. Both variants remained perfectly associated with the cataract phenotype in more than 500 animals (**Table 1**).

**Table:** **Table 1.** Association of coding variants with the cataract phenotype in Romagnola cattle.

Genotype	Cases	Obligate carriers^a^	Romagnola population controls^b^
ENSBTAG00000039845: c.687G>C			
G/G			535
G/C		5	36
C/C	4		
*NID1*: c.3579_3604+829del			
wt/wt			1012
wt/del		5	45
del/del	4		

a Parents of affected offspring were classified as obligate carriers.

b A larger cohort was genotyped for the *NID* variant to evaluate the frequency of the defect allele within the breed.

The *NID1* gene encodes a protein which is involved in matrix assembly and is expressed in various tissues and reportedly in lenses [30], [31]. A protein sequence based database search for the uncharacterized locus *ENSBTAG00000039845* revealed 70% sequence coverage and 57% sequence identity with the putative PRAME family member 24-like protein of *Bos taurus* (XP_606544.2). In addition, the result pointed out 63% sequence coverage and 98% identity with putative PRAME family member 25-like protein in *Bos mutus* (XP_005905668.1). PRAME family members are encoded by genes which are primarily involved in immunity and reproduction, and whose expression is restricted to the testis and a variety of cancers [32]. As an experimental control, we extracted RNA from skin and lenses of healthy cattle. RT-PCR revealed no evidence for expression of the *ENSBTAG00000039845* transcript neither in the skin nor in the lens, but interestingly, we observed *NID1* expression in both skin and lens (**Figure S2**). Thus we concluded that the *NID1* deletion is much more likely to cause cataract than the *ENSBTAG00000039845* variant. The *NID1* deletion consisting in the loss of hundreds of bases was potentially more disruptive compared to the exchange of a single less conserved amino acid in the uncharacterized locus which is not expressed in the lens.

None of the 1062 healthy Romagnola cattle genotyped for the *NID1* deletion had the homozygous mutant genotype, but 50 of them were determined to be carriers. All available ancestors of the 4 cases were shown to be carriers including the common founder sire *Marte* confirming the assumed recessive inheritance (**Figure 2**). In addition, a complete pedigree of all carriers with available records indicating that the mutation occurs restricted to a certain breeding line of Romagnola cattle was built (**Figure S3**). The mutation occurred at least two generations before the bull *Marte* thus confirming the initial suspicion that a recent mutation event in a common ancestor was responsible for the outbreak. The frequency of the deleterious *NID1* allele within this large sample of Romagnola cattle was 2.4%. This is quite low in comparison to recently determined frequencies for other recessive defects in Italian beef cattle [33], [34] and might explain the fact that until know no further affected animals have been observed.

**Figure 5:**
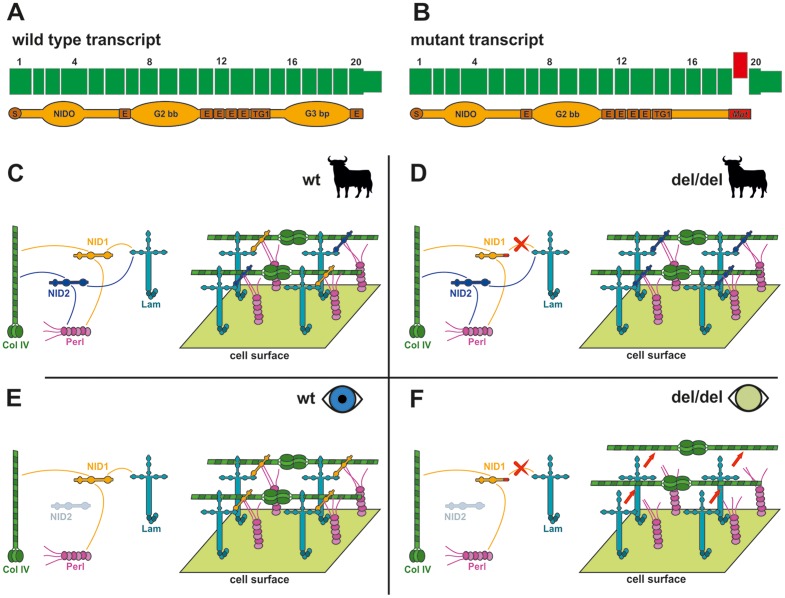
Tissue-specific inferred pathological mechanism. (**A**) Wild type transcript. The translated NID1 protein is shown. *S*: Signal peptide; *NIDO*: Nidogen domain; *E*: EGF-like domains *G2bb*: G2 beta-barrel domain; G3bp: G3 beta-propeller domain, binding laminin [36] (**B**) Mutated NID1. The skipped exon is represented in red. The C-terminal part of the protein is altered severely and part of the G3 domain is lost. (**C**) Matrix assembly in various body tisses of an animal (like muscle, heart, kidney, skin, [31]) with wild type NID; NID1 and NID2 are both expressed in detectable level; Both interact functionally with laminin and participate to the matrix assembly, probably compensating each for the lack of the other [41]–[45]. (**D**) Body tissues of an animal lacking a functional NID1: in all the tissue in which NID2 is expressed (all barring brain and lenses according to literature [31]) NID2 compensates for the lack of NID1 [45]–[47]. (**E**) Eye of an individual with functional NID1. NID2 is not expressed at detectable levels, but NID1 alone is enough in its role of bridge protein in the matrix assembly. (**F**) Eye of an individual lacking of functional NID1. NID1 cannot interact functionally with laminin, and this defect is not compensated in the lens tissue, leading to matrix instability and ultimately to a pathological condition. (**C-F**) Simplified version of basal membrane [38]. *NID1*: Nidogen 1. *NID2*: Nidogen 2. *Lam*: Laminins. *Col IV*: Collagen IV. *Perl*: Perlecan. Only interactions of the Nidogens with other elements are shown. Nidogens role is to bridge the interactions among the main matrix proteins [36].

The effect of the deletion on the *NID1* transcript was investigated with cDNA prepared from the skin tissue of an affected animal homozygous for the genomic deletion and a healthy control homozygous for the wild type allele. Sequencing of an RT-PCR product containing the exon 19- exon 20 junction showed that mRNA produced in the case, but not the control, lacked the entirety of exon 19 (**Figure 5**). This exon skipping results in a mutant transcript containing a frame-shift which predicts a truncation of 50 amino acids and contain 27 amino acids different from those found in the wild type protein (**Figure S4**). This predicts deletion of a portion of the G3 beta propeller domain which binds to laminin, and to the sixth EGF-like domain in the C-terminal part of the protein [35]. If not nonsense-mediated decay prevents transcription this would ultimately severely damage the role of *NID1* in the matrix assembly (**Figure 5**) [36].

### Role of *NID1* during cataractogenesis

In mammals are two known multivalent basement membrane (BM) binding proteins of the nidogen family called NID1 and NID2 [37]–[40] Basement membranes are found between cell surfaces and the interstitium or between cells [41]. Pathological conditions can arise due to mutations which can disrupt the extracellular matrix and/or its linkages leading to the loss of matrix integrity, adhesion strength and/or receptor-mediated signaling, affecting nerves, muscles, skin, kidneys and various tissues [42]. Interactions of the NID1 and NID2 proteins with many other matrix components have been demonstrated *in vitro*, in particular with laminins and collagen IV having the role of a stabilizing bridge (**Figure 5**) [40], [43], [44]. Deletion of either *NID1* or *NID2* did not reveal alterations in the BM architecture [31], [45], [46] and it has been suggested that the two proteins may be partially redundant, each compensating for the contingent lack of the other [45], [47]; their tissue-specificity in development has been suggested [31], [48], [49]. Nonetheless, mice lacking both nidogens die shortly after birth from lung and heart anomalies, which are directly correlated to BM defects [50]. *Nid1* knockout mice showed differences in some of crystal lenses of 7-week-old *Nid1* null mice compared to wild type [31]. In detail, they described the border between elongating fiber cells and the posterior lens capsule as being highly irregular [31]. In the same study the authors reported that, in the brain and eye lenses, the intrinsic expression level of *Nid2* seems to be lower as compared to other tissues in wild-type mice. No obvious up-regulation of *Nid2* was detected in *Nid1* -/- mice using western blot [31]. These authors suggested that both *Nid1* and *Nid2* can generally compensate for the non-functionality of the other. Nonetheless, the tissue-specific expression of a single variant can trigger a pathological condition if this variant, being the only one expressed in that tissue, is not functional (**Figure 5**). To verify if this is also the case in cattle, we investigated the presence of *NID1* and *NID2* transcript in normal bovine lenses compared to skin. RT-PCR indeed showed detectable level of *NID2* transcript in crystal lenses while in skin both *NID1* and *NID2* transcripts were expressed (**Figure S2**). We therefore concluded that the pathological condition is circumscribed to the eye (and possibly nervous tissue) because of tissue-specific expression of the *NID1* and *NID2* genes (**Figure 5**). One of the affected calves in our study showed an abnormal, albeit light, head movement (**Video S1**). Interestingly, head bobbing and seizure-like behavior have been reported in *Nid1* knockout mice, more severe if one *Nid2* allele was mutated as well [31], [51]. Furthermore, we found no evidence for the presence of other reported accompanied alterations affecting limb development [52]. This could suggest that, since the signs in our animals were less pronounced, it was possible that the deletion of larger portions of the protein could lead to a more severe condition, and, perhaps, this could also suggest that, in our calves, the altered *NID1* mRNA was translated into a shorter, but not completely defective, polypeptide. Gresham et al. have shown that different domains of NID1 activate distinct functions such as chemotaxis or phagocytosis [53]. Recently, a probably dominant acting *de novo* nonsense *NID1* mutation in a family with Dandy-Walker syndrome and occipital cephaloceles resulting in in the loss of the entire G2 and G3 regions of NID1 protein including all the EGF-like domains has been reported [54]. In our study the loss of the NID G3 domain is obviously not sufficient to create a pathological condition in heterozygous animals. Therefore, it is reasonable to assume that the milder phenotype observed in cattle could be due to the location of the mutation concerning only the terminal part of the protein. Overall, it appears that *NID* mutations can lead to very diverse phenotypes in mammals.

Because of the possible role played by *NID1* in eye lenses, Dong and colleagues actually suggested that *NID1* knockout mice could be used as a model system to further explore the role of the lens capsule in the cellular organization of the crystalline lens [31]. Still, the alterations reported are unclear, and there are authors reporting no alterations at all in crystal lenses [50]. In addition, the role of NID1 in signs other than those of a neurological nature is often overlooked [55], [56]. In his paper, May observed alterations in the inner limiting membrane in the posterior eye segment of *NID1* -/- mice [57]. The author speculated that specific mechanical forces and eye size/proportion can play a role in the stress suffered by basement membranes, with NID1 being important to their stability [57]. Along the same lines, it could therefore be speculated that our cataract phenotype in cattle, caused by nonfunctional NID1, could be, at least in part, due to the anatomical differences in the bovine eye as compared to the murine eye. Following this line of thinking, it could then be asked whether mice would accurately model the eye membrane alteration of large mammals (humans included), at least the alterations involving basal membrane stability. Indeed, it has previously been reported that murine models, although being very important in the study of eye lens pathologies, are far from being related to their human counterparts with regard to genotype/phenotype correlation [58]. In our study, it can be verified that cataractic lenses in cattle with nonfunctional NID1 were detectable immediately and clearly macroscopically.

### Conclusions

Our findings add the *NID1* gene to the list of candidates for nuclear cataract. In addition, the identification of a naturally occurring mutation for cataract in Romagnola cattle provides an interesting large animal model for human cataract. This study highlights the potential of the unique family structures of livestock populations in combination with state-of-the-art methods in order to determine causative mutations for a recessive defect in a short term. Furthermore, it was shown that, if reported and studied, a usually unreported condition, could possibly give to novel insights. To our knowledge, this is the first recorded causative mutation for recessive inherited cataract in cattle. It is the first time that *NID1* has been associated with crystal lens alteration in bigger mammals and a confirmation of the crucial involvement of *NID1* in crystal lens integrity as has previously been suggested in mice.

## Material and Methods

### Ethics Statement

All handling of the animals was conducted according to national and international guidelines for animal welfare. There is no permit number as this study was not based on an invasive animal experiment and used naturally occurring cases. This is a very special situation in veterinary medicine, as the data is from client-owned cattle which underwent veterinary exams; there was no “animal experiment” according to the legal definition in Italy. The samples used were taken a single cattle farm in Italy and the cattle owner agreed that the samples could be used in the study. Skin samples were taken from punches needed to clip the code ID numbers to the animal ears. Finally, the unaffected eye lenses came from routine necropsies on cattle with unrelated conditions which were carried out in the Department of Veterinary Medical Sciences of the University of Bologna.

### Animals and SNP genotyping

Blood samples were collected from 4 affected calves and their parents from the same farm. Genotyping of these cases was performed using the BovineHD BeadChip (Illumina), including 777,961 evenly distributed SNPs and standard protocols as recommended by the manufacturer. In addition, stored DNA from 1057 healthy Romagnola cattle was also used, resulting in a total of 1066 samples from this breed.

### Genome-wide association and homozygosity mapping

The GenAbel package in R studio was used for GWAS [59]. As a preliminary step in the analysis a first quality control to remove markers and individuals with call rates <90% from the analysis was carried out. Markers with minor allele frequency <5% were removed and also markers strongly deviating from the Hardy-Weinberg equilibrium (p<10^−6^). This was followed by a mixed model association study. The threshold of P≤0.01 for genome-wide significance was Bonferroni-adjusted to account for multiple testing (0.01/549,341 = 1.82×10^−8^). PLINK software [60] was used to search for extended intervals of homozygosity with shared alleles as described previously. Individuals and SNPs were selected using the commands –keep, and –extract while final files were generated through the –merge command. Homozygosity analysis was carried out on all cases using the commands –cow, –homozyg and –homozyg-group.

### Whole genome re-sequencing and variant calling

We prepared a fragment library with a 300 bp insert size and collected one lane of Illumina HiSeq2500 paired-end reads (2×100 bp); the fastq files were created using Casava 1.8. We obtained a total of 356,094,795 paired-end reads which were then mapped to the cow reference genome UMD3.1/bosTau6 and aligned using Burrows-Wheeler Aligner (BWA) version 0.5.9-r16 [61] with default settings. The mapping showed 313,195,442 reads had unique mapping positions. The SAM file generated by BWA was then converted to BAM and the reads sorted by chromosome using samtools [62]. PCR duplicates were marked using Picard tools (http://sourceforge.net/projects/picard/). We used the Genome Analysis Tool Kit (GATK version 2.4.9, [63]) to perform local realignment and to produce a cleaned BAM file. The genome data has been made freely available under accession no. PRJEB5965 at the European Nucleotide Archive [64].

Variant calls were then made with the unified genotyper module of GATK. The variant data for each sample was obtained in variant call format (version 4.0) as raw calls for all samples and sites flagged using the variant filtration module of GATK. Variant filtration was performed, following best practice documentation of GATK version 4. The snpEFF software [65] together with the UMD3.1/bosTau Ensembl annotation was used to predict the functional effects of detected variants. The Delly package was used to detect structural variants in cleaned BAM files. Delly uses variation in pair-end reads distance and orientation to find deletions, duplications, inversions and translocations. Structural variation software that are based on coverage and orientation are unable to detect variations larger than the insert size, as read mapping software usually requires the library insert size as an argument for aligning within range. Hence, in order to avoid missing large inserts, deletions and false positives all detected variants in the candidate region were also manually inspected [29].

### Sanger sequencing and genotyping

The associated variants were genotyped by re-sequencing of targeted PCR products using Sanger sequencing technology. PCR primers were designed using PRIMER3 [66]. PCR products were run on 0.8% agarose gel, 0.5 µg/ml ethidium bromide. PCR products were amplified using flanking primers (*NID1* (F) TCCAAGCGACAAAAGAGGTT, (R) TTTCCGCTCGATACAGTCAA; *ENSBTAG00000039845* (F) TGTGGCTCCTAAATGACCAA, (R) ACTTGGAGGATCCCAGGACT; *NID1* cDNA (F) TTTCCGCTCGATACAGTCAA, (R) CTTGAAGGGCTGCAGCC with AmpliTaqGold360Mastermix (Life Technologies) and the products directly sequenced using the PCR primers on an ABI 3730 capillary sequencer (Life Technologies) after treatment with exonuclease I (N.E.B) and rAPid alkaline phosphatase (Roche). Sequence data were analyzed using Sequencher 5.1 (GeneCodes). We used an alternative fragment size analysis assay to genotype additional cattle for the *NID1* deletion using three primers: (F) CATCAGGGAAATCCTGCTGT and (R_wild type_) CAGGTGGGTTACCTTCAGGA, and (R_mutant_) GTGACCTGGAAAAGGCAGAA. Products were visualized on an ABI 3730 capillary sequencer and analyzed with the GeneMapper 4.0 software (Life Technologies). Fragments with the deletion are 286 bp in length and fragments without the deletion are 176 bp in length.

### RNA extraction and cDNA product amplification

The RNA was extracted from skin tissue and crystal lenses with the same procedure using RNeasy mini kit (Qiagen). First, the tissue was finely crushed in TRIZOL (Ambion) using mechanical means, chloroform was then added and the RNA separated through centrifugation. Additional passages were carried out as described by the manufacturer. Genomic DNA contamination was eliminated enzymatically using the Quantitect Reverse Transcription Kit (Qiagen). The same kit was used to synthetize cDNA, as described by the manufacturer. The cDNA fragments of target genes PCR products were amplified using the following primers: *NID1*: (F) CTTGAAGGGCTGCAGTATCC, (R) TTTCCGCTCGATACAGTCAA; *NID2* (F) GACTCAGCTGTGACCTGCAC, (R) AAGACCCGGTCAACATTCAG; *ENSBTAG00000039845*: (F) TGTGGCTCCTAAATGACCAA, (R) ACTTGGAGGATCCCAGGACT; *GADPH* (F) ACCCAGAAGACTGTGGATGG, (R) CCAGCAGCTGAGGAACTCTT. The PCRs were carried out with AmpliTaqGold360Mastermix (Life Technologies). The PCR products were run on 2% agarose gel and 0.5 µg/ml ethidium bromide.

## Supporting Information

Figure S1 **QQ-plot.** QQ- plots showing the observed versus expected -log p-values. The diagonal line in the QQ plots indicates the distribution of SNP markers under the null hypothesis, and the skewing of a marker toward the upper side suggests that it has a stronger association with the pathological condition than one would expected by mere chance. The deviation of observed values from the expected is clearly visible and indicates a consistent difference between cases and controls and reflects the GWAS result obtained.
(TIF)

Figure S2 **Tissue specific gene expression.** The picture shows the amplified fragment of cDNA extracted from skin (left) and crystal lenses (right) of healthy cattle. *GADPH*: Glyceraldehyde 3-phosphate dehydrogenase. The samples are from 1-year old calves slaughtered *NID1*: nidogen-1; *NID2*: Nidogen-2; *PRAME*: uncharacterized locus *ENSBTAG00000039845*; Mk: 100 bp ladder. Note that the PRAME like transcript is absent in both skin and crystal lens and that the *NID2* transcript is absent in the crystal lens albeit to skin.
(TIF)

Figure S3 **Extended family tree.** The family tree in Figure 2 expanded adding all the carriers detected within the population whose the ancestry was known and able to be reconstructed. Males are represented by squares, females by circles. Affected animals are shown with fully black symbols and genotyped carriers with a half-filled symbol. All animals with empty symbols were not available for genotyping. The purpose of this picture is to show the recessive inheritance of the mutation and to find a common ancestor. Note how most animals show having *Marte* as a common ancestor. *Marte* shares common ancestry with a number of carriers in *Emiro*, son of *Titano* and ancestor of another carrier.
(TIF)

Figure S4 **NID protein sequences.** Predicted amino acid sequence of the wild type and mutated NID1 protein. In green fragment of the protein present only in the wild type, in red the predicted change in the mutant.
(PDF)

Table S1 **Top 14 significant SNPs.**
(PDF)

Table S2 **Region of homozygosity among the affected animals.**
(PDF)

Table S3 **List of all the variants in the BTA 28 candidate region.** Non-synonymous variants are bolded.
(PDF)

Video S1 **Neurological abnormalities in a single cataract case.**
(MP4)

## References

[1] Koenigsberg R (1989) Churchill's Medical Dictionary. Churchill Livingstone Inc. 308 p.

[2] Deng H, Yuan L (2014) Molecular genetics of congenital nuclear cataract. Eur J Med Genet 57: 113–122. 24384146 10.1016/j.ejmg.2013.12.006

[3] Yi J, Yun J, Li ZK, Xu CT, Pan BR (2011) Epidemiology and molecular genetics of congenital cataracts. Int J Ophthalmol 4: 422–432. 22553694 10.3980/j.issn.2222-3959.2011.04.20 PMC3340856

[4] Mesa R, Bassnett S (2013) UV-B-induced DNA damage and repair in the mouse lens. Invest Ophthalmol Vis Sci 54: 6789–6797. 24022010 10.1167/iovs.13-12644 PMC3799563

[5] Sinha R, Kumar C, Titiyal JS (2009) Etiopathogenesis of cataract: Journal review. Indian J Ophthalmol 2009 57: 245–249.

[6] Petrash JM (2013) Aging and age-related diseases of the ocular lens and vitreous body. Invest Ophthalmol Vis Sci 54: ORSF54–59. 24335070 10.1167/iovs.13-12940 PMC3864378

[7] Chew EY (2013) Nutrition effects on ocular diseases in the aging eye. Invest Ophthalmol Vis Sci 54: ORSF42–47. 24335067 10.1167/iovs13-12914 PMC4154321

[8] Churchill A (2011) GrawJ (2011) Clinical and experimental advances in congenital and paediatric cataracts. Philos Trans R Soc Lond B Biol Sci 366: 1234–1249. 21402583 10.1098/rstb.2010.0227 PMC3061104

[9] Hejtmancik JF (2008) Congenital cataracts and their molecular genetics. Semin Cell Dev Biol 19: 134–149. 18035564 10.1016/j.semcdb.2007.10.003 PMC2288487

[10] He W, Li S (2000) Congenital cataracts: gene mapping. Hum Genet 106: 1–13. 10982175 10.1007/s004390051002

[11] Huang B, He W (2010) Molecular characteristics of inherited congenital cataracts. Eur J Med Genet 53: 347–357. 20624502 10.1016/j.ejmg.2010.07.001

[12] Liang J (2004) Interactions and chaperone function of A-crystallin with T5PgC-crystallin mutant. Protein Sci 13: 2476–2482. 15322286 10.1110/ps.04815104 PMC2280011

[13] Shiels A, Bennett TM, Hejtmancik JF (2010) Cat-Map: putting cataract on the map. MolVis 16: 2007–2015. PMC2965572 21042563

[14] Liu B, Song S, Hanson H, Liang J (2008) Protein-protein interactions involving congenital cataract T5P γC-crystallin mutant: a confocal fluorescence microscopy study. Exp Eye Res 87: 515–520. 18926820 10.1016/j.exer.2008.08.021 PMC2644446

[15] Talla V, Narayanan C, Srinivasan N, Balasubramanian D (2006) Mutation causing self-aggregation in human γC-crystallin leading to congenital cataract. Invest Ophthalmol Vis Sci 47: 5212–5217. 17122105 10.1167/iovs.06-0427

[16] Hunter LS, Sidjanin DJ, Johnson JL, Zangerl B, Galibert F, et al (2006) Radiation hybrid mapping of cataract genes in the dog. Mol Vis 12: 588–596. 16760895 PMC1509099

[17] Gelatt KN, Das ND (1984) Animal models for inherited cataracts: a review. Curr Eye Res 3: 765–778. 6734258 10.3109/02713688409065600

[18] Tripathi BJ, Tripathi RC, Borisuth NS, Dhaliwal R, Dhaliwal D (1991) Rodent models of congenital and hereditary cataract in man. Lens Eye Toxic Res 8: 373–413. 1958636

[19] Wilson GR, Morton JD, Palmer DN, McEwan JC, Gately K, et al (2012) The locus for an inherited cataract in sheep maps to ovine chromosome 6. Mol Vis 18: 1384–1394. 22690116 PMC3370893

[20] Morton JD, Lee HY, McDermott JD, Robertson LJ, Bickerstaffe R, et al (2013) Macrocyclic calpain inhibitor slows the development of inherited cortical cataracts in a sheep model. Invest Ophthalmol Vis Sci 54: 389–395. 23211821 10.1167/iovs.12-11088

[21] Detlefson JA, Yapp WW (1920) The inheritance of congenital cataract in cattle. Amer Nat 54: 277–280.

[22] Gregory PW, Mead SW, Regan WM (1943) A congenital hereditary eye defect of cattle. J Hered 34: 125–128.

[23] Krump L, O'Grady L, Lorenz I, Grimes T (2014) Congenital cataracts in an Ayrshire herd: a herd case report. Ir Vet J 67: 2. 24460638 10.1186/2046-0481-67-2 PMC3905159

[24] Hässig M, Jud F, Naegeli H, Kuppe J, Spiess M (2009) Prevalence of nuclear cataract in Swiss veal calves and its possible association with mobile telephone antenna base stations. Schweiz Archiv Tierheilkd 151: 471–478. 10.1024/0036-7281.151.10.471 19780007

[25] Hirano T, Matsuhashi T, Kobayashi N, Watanabe T, Sugimoto Y (2012) Identification of an FBN1 mutation in bovine Marfan syndrome-like disease. Anim Genet 43: 11–17. 22221020 10.1111/j.1365-2052.2011.02209.x

[26] Testoni S, Bartolone E, Rossi M, Patrignani A, Bruggmann R, et al (2012) KDM2B is implicated in bovine lethal multi-organic developmental dysplasia. PLoS One 7: e45634. 23029151 10.1371/journal.pone.0045634 PMC3459949

[27] Murgiano L, Sacchetto R, Testoni S, Dorotea T, Mascarello F, et al (2012) Pseudomyotonia in Romagnola cattle caused by novel ATP2A1 mutations. BMC Vet Res 8: 186. 23046865 10.1186/1746-6148-8-186 PMC3545862

[28] Clark JI, Matsushima H, David LL, Clark JM (1999) Lens cytoskeleton and transparency: a model. Eye (Lond) 13: 417–424. 10627819 10.1038/eye.1999.116

[29] Rausch T, Zichner T, Schlattl A; Stütz AM, Benes V, et al (2012) DELLY: structural variant discovery by integrated paired-end and split-read analysis. Bioinformatics 28: i333–i339. 22962449 10.1093/bioinformatics/bts378 PMC3436805

[30] Salmivirta K, Talts JF, Olsson M, Sasaki T, Timpl R, et al (2002) Binding of mouse nidogen-2 to basement membrane components and cells and its expression in embryonic and adult tissues suggest complementary functions of the two nidogens. Exp Cell Res 279: 188–201. 12243745 10.1006/excr.2002.5611

[31] Dong L, Chen Y, Lewis M, Hsieh JC, Reing J, et al (2002) Neurologic defects and selective disruption of basement membranes in mice lacking entactin-1/nidogen-1. Lab Invest 82: 1617–1630. 12480912 10.1097/01.lab.0000042240.52093.0f

[32] Chang TC, Yang Y, Yasue H, Bharti AK, Retzel EF (2011) The expansion of the PRAME gene family in Eutheria. PLoS One 6: e16867. 21347312 10.1371/journal.pone.0016867 PMC3037382

[33] Murgiano L, Testoni S, Drögemüller C, Bolcato M, Gentile A (2013) Frequency of bovine congenital pseudomyotonia carriers in selected Italian Chianina sires. Vet J 195: 238–240. 22627047 10.1016/j.tvjl.2012.04.021

[34] Murgiano L, Drögemüller C, Sbarra F, Bolcato M, Gentile A (2014) Prevalence of paunch calf syndrome carriers in Italian Romagnola cattle. Vet J 200: 459–461. 24792452 10.1016/j.tvjl.2014.03.020

[35] Darbro BW, Mahajan VB, Gakhar L, Skeie JM, Campbell E, et al (2013) Mutations in extracellular matrix genes NID1 and LAMC1 cause autosomal dominant Dandy-Walker malformation and occipital cephaloceles. Hum Mutat 34: 1075–1079. 23674478 10.1002/humu.22351 PMC3714376

[36] Takagi J, Yang Y, Liu JH, Wang JH, Springer TA (2003) Complex between nidogen and laminin fragments reveals a paradigmatic beta-propeller interface. Nature 424: 969–974. 12931195 10.1038/nature01873

[37] Carlin B, Jaffe R, Bender B, Chung AE (1981) Entactin, a novel basal lamina-associated sulfated glycoprotein. J Biol Chem 256: 5209–5214. 6262321

[38] Timpl R, Dziadek M, Fujiwara S, Nowack H, Wick G (1983) Nidogen: a new, self-aggregating basement membrane protein. Eur J Biochem 137: 455–465. 6420150 10.1111/j.1432-1033.1983.tb07849.x

[39] Kimura N, Toyoshima T, Kojima T, Shimane M (1998) Entactin-2: a new member of basement membrane protein with high homology to entactin/nidogen. Exp Cell Res 241: 36–45. 9633511 10.1006/excr.1998.4016

[40] Kohfeldt E, Sasaki T, Göhring W, Timpl R (1998) Nidogen-2: a new basement membrane protein with diverse binding properties. J Mol Biol 282: 99–109. 9733643 10.1006/jmbi.1998.2004

[41] Yurchenco PD, Amenta PS, Patton BL (2004) Basement membrane assembly, stability and activities observed through a developmental lens. Matrix Biol 22: 521–538. 14996432 10.1016/j.matbio.2003.10.006

[42] Yurchenco PD, Patton BL (2009) Developmental and pathogenic mechanisms of basement membrane assembly. Curr Pharm Des 15: 1277–1294. 19355968 10.2174/138161209787846766 PMC2978668

[43] Fox J, Mayer U, Nischt R, Aumailley M, Reinhardt D, et al (1991) Recombinant nidogen consists of three globular domains and mediates binding of laminin to collagen type IV. EMBO J 10: 3137–3146. 1717261 10.1002/j.1460-2075.1991.tb04875.x PMC453035

[44] Aumailley M, Battaglia C, Mayer U, Reinhardt D, Nischt R, et al (1993) Nidogen mediates the formation of ternary complexes of basement membrane components. Kidney Int 43: 7–12. 8433572 10.1038/ki.1993.3

[45] Murshed M, Smyth N, Miosge M, Karolat J, Krieg T, et al (2000) The absence of nidogen-1 does not affect murine basement membrane development. Mol Cell Biol 20: 7007–7012. 10958695 10.1128/mcb.20.18.7007-7012.2000 PMC88775

[46] Schymeinsky J, Nedbal S, Miosge N, Pöschl E, Rao C, et al (2002) Gene structure and functional analysis of the mouse nidogen-2 gene: nidogen-2 is not essential for basement membrane formation in mice. Mol Cell Biol 22: 6820–6830. 12215539 10.1128/MCB.22.19.6820-6830.2002 PMC135501

[47] Miosge N, Sasaki T, Timpl R (2002) Evidence of nidogen-2 compensation for nidogen-1 deficiency in transgenic mice. Matrix Biol 21: 611–621.. 12475645 10.1016/s0945-053x(02)00070-7

[48] Miosge N, Holzhausen S, Zelent C, Sprysch P, Herken R (2001) Nidogen-1 and nidogen-2 are found in basement membranes during human embryonic development. Histochem J 33: 523–530. 12005023 10.1023/a:1014995523521

[49] Neu R, Adams S, Munz B (2006) Differential expression of entactin-1/nidogen-1 andentactin-2/nidogen-2 in myogenic differentiation. Differentiation 74: 573–582. 17177854 10.1111/j.1432-0436.2006.00100.x

[50] Bader B, Smyth N, Nedbal S, Miosge N, Baranowsky A, et al (2005) Compound genetic ablation of nidogen 1 and 2 causes basement membrane defects and perinatal lethality in mice. Mol Cell Biol 25: 6846–6856. 16024816 10.1128/MCB.25.15.6846-6856.2005 PMC1190363

[51] Vasudevan A, Ho MS, Weiergräber M, Nischt R, Schneider T (2010) Basement membrane protein nidogen-1 shapes hippocampal synaptic plasticity and excitability. Hippocampus 20: 608–620. 19530222 10.1002/hipo.20660

[52] Böse K, Nischt R, Page A, Bader BL, Paulsson M, et al (2006) Loss of nidogen-1 and -2 results in syndactyly and changes in limb development. J Biol Chem 281: 39620–39629. 17023412 10.1074/jbc.M607886200

[53] Gresham HD, Graham IL, Griffin GL, Hsieh JC, Dong LJ, et al (1996) Domain-specific interactions between entactin and neutrophil integrins: G2 domain ligation of integrin alpha3beta1 and E domain ligation of the leukocyte response integrin signal for different responses. J Biol Chem 271: 30587–30594. 8940031 10.1074/jbc.271.48.30587

[54] Darbro BW, Mahajan VB, Gakhar L, Skeie JM, Campbell E, et al (2013) Mutations in extracellular matrix genes NID1 and LAMC1 cause autosomal dominant Dandy-Walker malformation and occipital cephaloceles. Hum Mutat 34: 1075–1079. 23674478 10.1002/humu.22351 PMC3714376

[55] Aszódi A, Legate KR, Nakchbandi I, Fässler R (2006) What mouse mutants teach us about extracellular matrix function. Annu Rev Cell Dev Biol 22: 591–621. 16824013 10.1146/annurev.cellbio.22.010305.104258

[56] Breitkreutz D, Koxholt I, Thiemann K, Nischt R (2013) Skin Basement Membrane: The Foundation of Epidermal Integrity—BM Functions and Diverse Roles of Bridging Molecules Nidogen and Perlecan. BioMed Research Int DOI: 10.1155 10.1155/2013/179784 PMC3618921 23586018

[57] May CA (2012) Distribution of nidogen in the murine eye and ocular phenotype of the nidogen-1 knockout mouse. ISRN Ophthalmol doi:10.5402 10.5402/2012/378641 PMC3912598 24555126

[58] Marchitti SA, Bateman JB, Petrash JM, Vasiliou V (2008) Mouse Models of the Cornea and Lens: Understanding Ocular Disease. In: Tsonis PA, editor, animal models in eye research. Academic Press, Elsiever.

[59] Aulchenko YS, Ripke S, Isaacs A, van Duijn CM (2007) GenABEL: an R library for genome-wide association analysis. Bioinformatics 23: 1294–1296. 17384015 10.1093/bioinformatics/btm108

[60] Purcell S, Neale B, Todd-Brown K, Thomas L, Ferreira MAR, et al (2007) PLINK: a toolset for whole-genome association and population-based linkage analysis. Am J Hum Genet 81: 559–575. 17701901 10.1086/519795 PMC1950838

[61] Li H, Durbin R (2009) Fast and accurate short read alignment with Burrows-Wheeler transform. Bioinformatics 25: 1754–1760. 19451168 10.1093/bioinformatics/btp324 PMC2705234

[62] Hompage Samtools (2014). Available: http://samtools.sourceforge.net. Accessed 2014 Feb 27.

[63] McKenna A, Hanna M, Banks E, Sivachenko A, Cibulskis K, et al (2010) The genome analysis toolkit: a MapReduce framework for analyzing next-generation DNA sequencing data. Genome Res 20: 1297–1303. 20644199 10.1101/gr.107524.110 PMC2928508

[64] Homepage European Nucleotide Archive (2014). Available: http://www.ebi.ac.uk/ena/data/view/PRJEB5965. Accessed 2014 Jul 14.

[65] Cingolani P, Platts A, Coon M, Nguyen T, Wang L, et al (2012) A program for annotating and predicting the effects of single nucleotide polymorphisms, SnpEff: SNPs in the genome of Drosophila melanogaster strain w1118; iso-2; iso-3. Fly 6: 80–92. 22728672 10.4161/fly.19695 PMC3679285

[66] Untergrasser A, Cutcutache I, Koressaar T, Ye J, Faircloth BC, et al (2012) Primer3 - new capabilities and interfaces. Nucleic Acids Res 40: e115.. 22730293 10.1093/nar/gks596 PMC3424584

