# Supplementary figures and images for: Looking the Cow in the Eye: Deletion in the NID1 Gene Is Associated with Recessive Inherited Cataract in Romagnola Cattle
Source: PLoS One. 2014 Oct 27;9(10):e110628. doi: 10.1371/journal.pone.0110628 (PMC4210201; doi:10.1371/journal.pone.0110628)

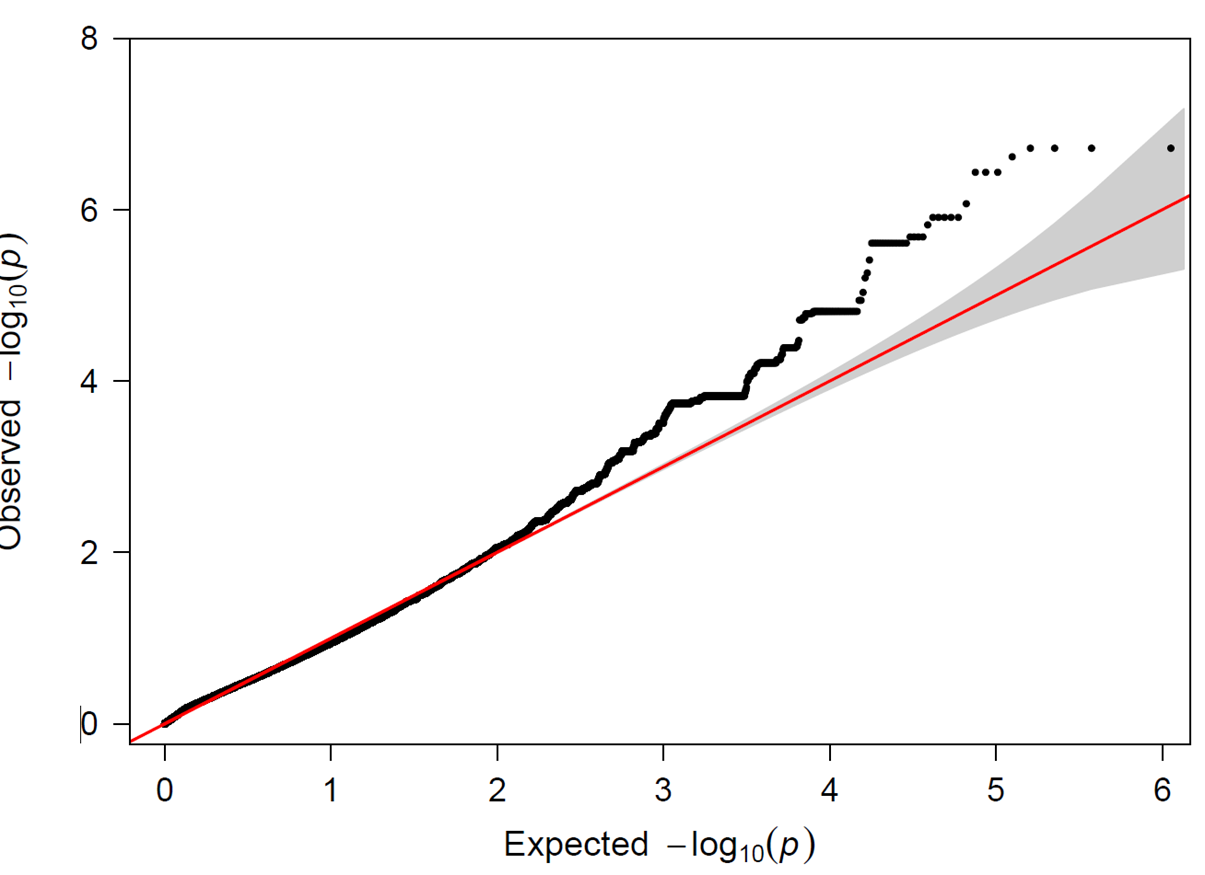

Supplement: Figure S1 — QQ-plot. QQ- plots showing the observed versus expected -log p-values. The diagonal line in the QQ plots indicates the distribution of SNP markers under the null hypothesis, and the skewing of a marker toward the upper side suggests that it has a stronger association with the pathological condition than one would expected by mere chance. The deviation of observed values from the expected is clearly visible and indicates a consistent difference between cases and controls and reflects the GWAS result obtained. (TIF) [file pone.0110628.s001.tif]

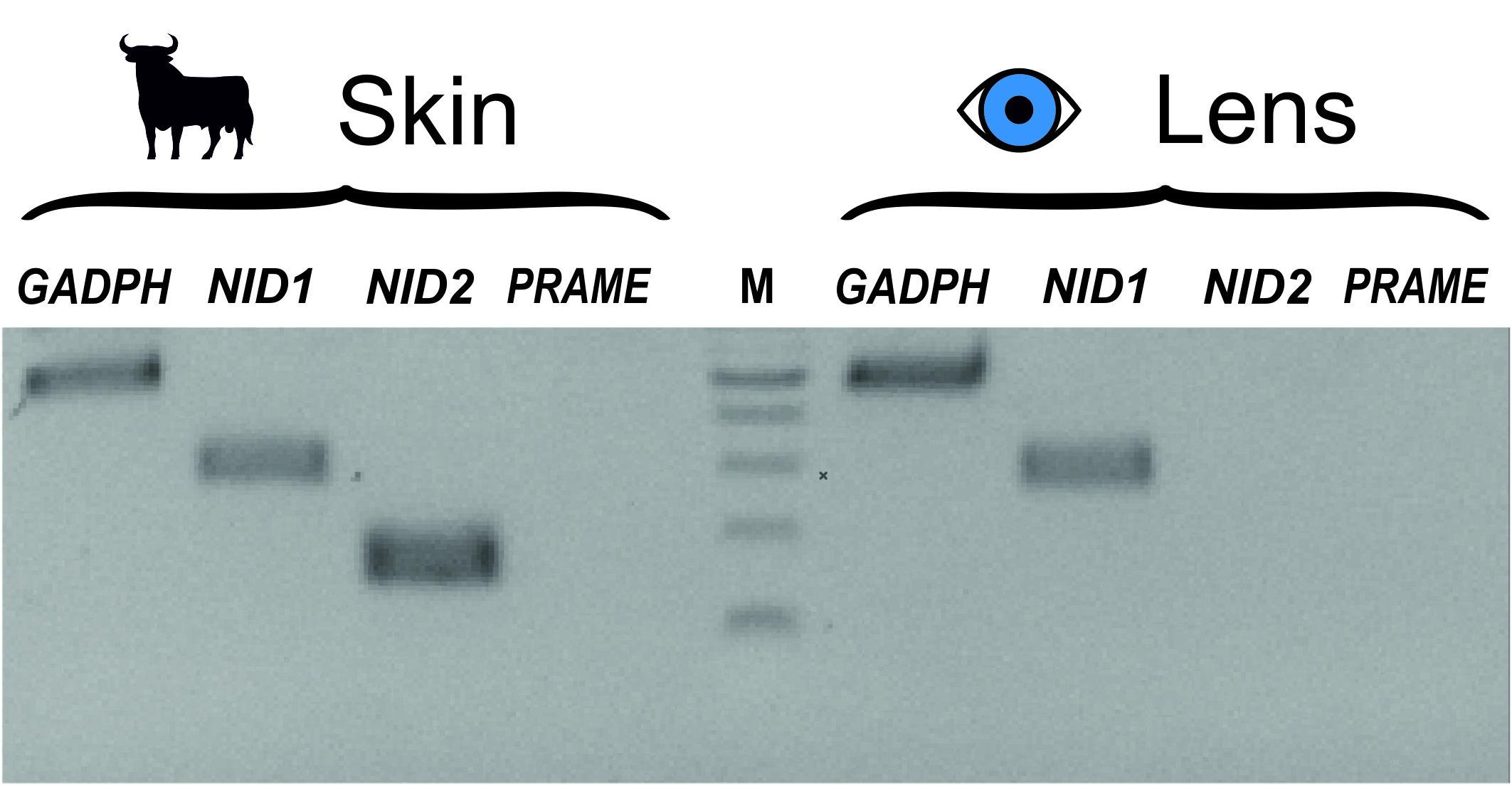

Supplement: Figure S2 — Tissue specific gene expression. The picture shows the amplified fragment of cDNA extracted from skin (left) and crystal lenses (right) of healthy cattle. GADPH: Glyceraldehyde 3-phosphate dehydrogenase. The samples are from 1-year old calves slaughtered NID1: nidogen-1; NID2: Nidogen-2; PRAME: uncharacterized locus ENSBTAG00000039845; Mk: 100 bp ladder. Note that the PRAME like transcript is absent in both skin and crystal lens and that the NID2 transcript is absent in the crystal lens albeit to skin. (TIF) [file pone.0110628.s002.tif]

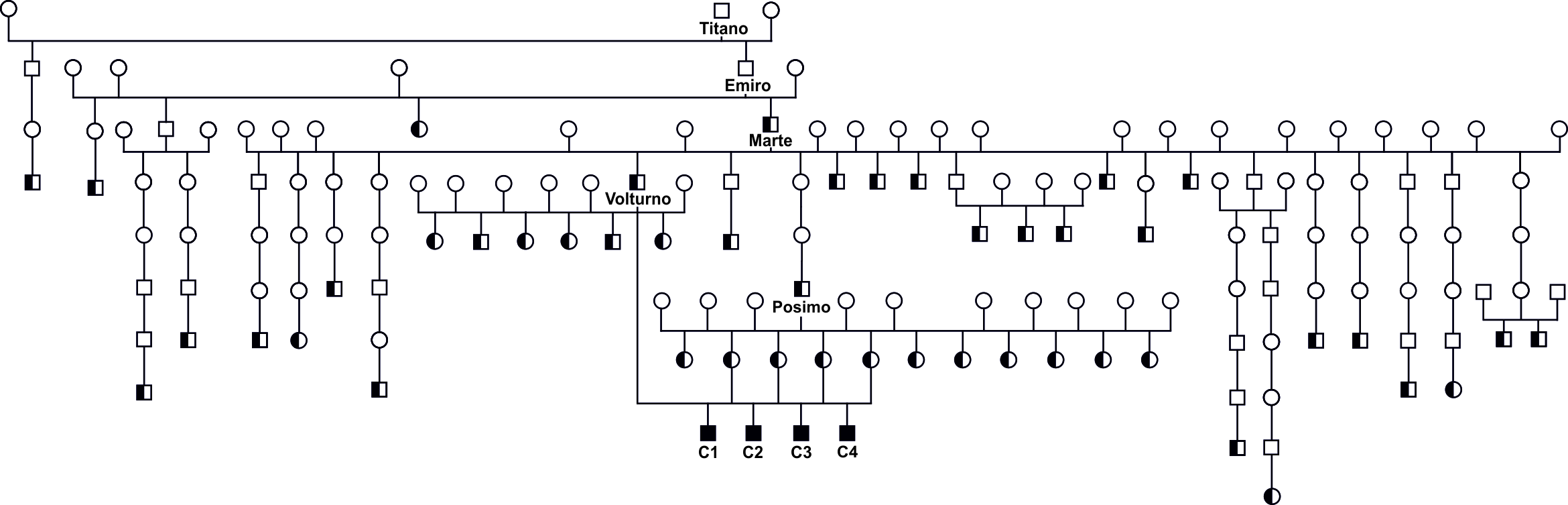

Supplement: Figure S3 — Extended family tree. The family tree in Figure 2 expanded adding all the carriers detected within the population whose the ancestry was known and able to be reconstructed. Males are represented by squares, females by circles. Affected animals are shown with fully black symbols and genotyped carriers with a half-filled symbol. All animals with empty symbols were not available for genotyping. The purpose of this picture is to show the recessive inheritance of the mutation and to find a common ancestor. Note how most animals show having Marte as a common ancestor. Marte shares common ancestry with a number of carriers in Emiro, son of Titano and ancestor of another carrier. (TIF) [file pone.0110628.s003.tif]
