# Supplementary material for: Looking the Cow in the Eye: Deletion in the NID1 Gene Is Associated with Recessive Inherited Cataract in Romagnola Cattle
Source: PLoS One. 2014 Oct 27;9(10):e110628. doi: 10.1371/journal.pone.0110628 (PMC4210201; doi:10.1371/journal.pone.0110628)
Supplement: Figure S4 — NID protein sequences. Predicted amino acid sequence of the wild type and mutated NID1 protein. In green fragment of the protein present only in the wild type, in red the predicted change in the mutant. (PDF) [file pone.0110628.s004.pdf]

Figure S4:

Wild-type Nidogen-1

MLATVRRSGAAWTRALLLQLLLAGPGGCLSRQELFPFGPEHGDLELEAGDDLVSPALELSTALHFFDRSDI
DSVYVTTNGIIAMSEPPAKETHPGPFPPTFGAVAPFLADLDTTDGLGKVYYREDLSPSVTQLAAECVQRGFP
EVSFKPSSAVVVTWESVAPYQGPSKDPTLEGKRNTFQSILASSDSSTYAIFLYPEDGLQFYTTFSKKEENQVP
AVVAFSQGLVGLIWKSDGAYNIFANDKESIGNLAKSSNSGLQGIWVFEIGSPATASGVVPADVNLGLDDGTE
YDDEDYDSVTRVGLEDAVTTSFPYEAPGRGDSGTYNTPSDLSPRRMATERPLTPPTEKTRSFQLPGERFPQQ
QPQVIDVDEVEETEIVFRYNTDSRQTCANNRHQCSVHAECRDFATGFCCRCVAGYTGNGRQCVAEGSPQR
VNGKVKGRIFVGDSQVPIVFENTDLHSYVVMNHGRSYTAISTIPETVGYSLLPLAPIGGIIGWMFAVEQDGF
KNGFSITGGEFTRQAEVTFVGHRDKLIIKQQFSGIDEHGHLTIDTELEGRVPQIAFGSSVHIEPYTELYHYSR
QVITSFSTREYTVTEPERHGTAPSHAHTYRWRQTITFRECLHDDSRPALPSTQQLSVDSVFVLYNQEERILR
YALSNSIGPVRDGSPDALQNPCYIGSHGCDSNAACRPGPGTQFTCECSIGFRGDGRTCYDIDECSEQPSVCG
NHAICNNHPGTFRCECVEGYQFSEAGTCVAAVGLRPVNHCETGLHDCDIPQRARCIYMGGSSYTCSCLPGF
SGDGRACQDVDECQPSRCHPDAFCYNTPGSFTCRCKSGYQGDGFHCVPGVVEKTRCQHEREHILGTADSS
RPRPPGLFVPECDEHGQYVPTQCHSSTGYCWCVDRDGREVQGTRTRSGMRPPCLSTVAPPVHYGPPVPTT
VIPLPPGTHLLFAQTGKIERLPLEGSTMTKSEAKTLLHAPGKVIIGLAFDCVDKMVYWTDISQPSIGRASLHG
GEPATIVRQDLGSPEGIALDHLGRNIFWTDSQLDRIEVAKLDGTQRRVLFETDLVNPRGIVTDSVRGNLYW
TDWNRDNPKIETSYMDGTNRRILVQDDLGLPNGLTFDAYSSQLCWVDAGAHRAECLKPGQSSRRKVLEGL
QYPFAVTSFGKNLYYTDWKTNSVVAVDLAVSKETDSFHPHKQTRLYGITSALSQCPEGHNYCSVNNGGCTH
LCLATPGSRTCRCPDNTLGVDCIERK

Mutant Nidogen-1

MLATVRRSGAAWTRALLLQLLLAGPGGCLSRQELFPFGPEHGDLELEAGDDLVSPALELSTALHFFDRSDI
DSVYVTTNGIIAMSEPPAKETHPGPFPPTFGAVAPFLADLDTTDGLGKVYYREDLSPSVTQLAAECVQRGFP
EVSFKPSSAVVVTWESVAPYQGPSKDPTLEGKRNTFQSILASSDSSTYAIFLYPEDGLQFYTTFSKKEENQVP
AVVAFSQGLVGLIWKSDGAYNIFANDKESIGNLAKSSNSGLQGIWVFEIGSPATASGVVPADVNLGLDDGTE
YDDEDYDSVTRVGLEDAVTTSFPYEAPGRGDSGTYNTPSDLSPRRMATERPLTPPTEKTRSFQLPGERFPQQ
QPQVIDVDEVEETEIVFRYNTDSRQTCANNRHQCSVHAECRDFATGFCCRCVAGYTGNGRQCVAEGSPQR
VNGKVKGRIFVGDSQVPIVFENTDLHSYVVMNHGRSYTAISTIPETVGYSLLPLAPIGGIIGWMFAVEQDGF
KNGFSITGGEFTRQAEVTFVGHRDKLIIKQQFSGIDEHGHLTIDTELEGRVPQIAFGSSVHIEPYTELYHYSR
QVITSFSTREYTVTEPERHGTAPSHAHTYRWRQTITFRECLHDDSRPALPSTQQLSVDSVFVLYNQEERILR
YALSNSIGPVRDGSPDALQNPCYIGSHGCDSNAACRPGPGTQFTCECSIGFRGDGRTCYDIDECSEQPSVCG
NHAICNNHPGTFRCECVEGYQFSEAGTCVAAVGLRPVNHCETGLHDCDIPQRARCIYMGGSSYTCSCLPGF
SGDGRACQDVDECQPSRCHPDAFCYNTPGSFTCRCKSGYQGDGFHCVPGVVEKTRCQHEREHILGTADSS
RPRPPGLFVPECDEHGQYVPTQCHSSTGYCWCVDRDGREVQGTRTRSGMRPPCLSTVAPPVHYGPPVPTT
VIPLPPGTHLLFAQTGKIERLPLEGSTMTKSEAKTLLHAPGKVIIGLAFDCVDKMVYWTDISQPSIGRASLHG
GEPATIVRQDLGSPEGIALDHLGRNIFWTDSQLDRIEVAKLDGTQRRVLFETDLVNPRGIVTDSVRGNLYW
TDWNRDNPKIETSYMDGTNRRILVQDDLGLPNGLTFDAYSSQLCWVDAGAHRAECLKPGQSSRRKVLEGL
QYPFAVTSFGKNLYYTDWKTSQLLLSEQRWLHPPLLGHPRKQDLPLP
