# Supplementary material for: Looking the Cow in the Eye: Deletion in the NID1 Gene Is Associated with Recessive Inherited Cataract in Romagnola Cattle
Source: PLoS One. 2014 Oct 27;9(10):e110628. doi: 10.1371/journal.pone.0110628 (PMC4210201; doi:10.1371/journal.pone.0110628)
Supplement: Table S1 — Top 14 significant SNPs. (PDF) [file pone.0110628.s005.pdf]

Table S1 – Top 14 significant SNPs

	Chromosome	Position*	chi2.1df**	P1df#	Pc1df##
**BovineHD4100018479**	28	6962697	41.68619	1.07E-10	1.78E-10
**BovineHD2800003023**	28	9889547	41.68619	1.07E-10	1.78E-10
**BovineHD2800003025**	28	9895373	41.68619	1.07E-10	1.78E-10
**BovineHD2800001993**	28	6673247	38.69312	4.96E-10	7.94E-10
**BovineHD2800002417**	28	8136855	36.37167	1.63E-09	2.54E-09
**BovineHD2800002989**	28	9781603	36.27947	1.71E-09	2.66E-09
**BovineHD2800002459**	28	8246055	36.17842	1.80E-09	2.80E-09
**BovineHD2800002463**	28	8256824	36.17842	1.80E-09	2.80E-09
**BovineHD2800003032**	28	9901827	36.15676	1.82E-09	2.83E-09
**BovineHD2800001012**	28	3323642	36.14479	1.83E-09	2.85E-09
**BovineHD2800003073**	28	10056945	34.25629	4.83E-09	7.34E-09
**BovineHD2800003074**	28	10061325	34.25629	4.83E-09	7.34E-09
**BovineHD2800002415**	28	8115821	34.15846	5.08E-09	7.71E-09
**BovineHD2800002987**	28	9767515	34.02109	5.45E-09	8.26E-09

*precise location of the SNP on the respective cattle chromosome.

**chi2.1df: Chi-square distribution of 1-d.f.

#P1df: corresponding list of P-values of 1-d.f. (additive or allelic) test for association between SNP and trait.

##Pc1df: P-values from the 1-d.f. test for association between SNP and trait (statistics is corrected for possible inflation); 1-d.f. = 1 degree of freedom.
