# Supplementary material for: Looking the Cow in the Eye: Deletion in the NID1 Gene Is Associated with Recessive Inherited Cataract in Romagnola Cattle
Source: PLoS One. 2014 Oct 27;9(10):e110628. doi: 10.1371/journal.pone.0110628 (PMC4210201; doi:10.1371/journal.pone.0110628)
Supplement: Table S2 — Region of homozygosity among the affected animals. (PDF) [file pone.0110628.s006.pdf]

Table S2 Homozygous regions

Chromosome	SNP1	SNP2	BP 1	BP 2	Kb
**3**	BovineHD0300009807	BovineHD0300025557	31235235	89181936	57946.7
**6**	BovineHD0600017644	BovineHD0600018930	63741074	68588167	4847.09
**7**	BovineHD0700014840	BovineHD0700015161	50959514	52464670	1505.16
**8**	BovineHD0800031812	BovineHD0800032314	106523016	107763189	1240.17
**28**	BovineHD2800000895	BovineHD2800003081	2870897	10073583	7202.69

Extended regions of homozygosity with simultaneous allele sharing among all the four cases. SNP1, SNP2: name of the flanking SNPs. BP1, BP2: base pair position of the SNP1 and SNP2, respectively. Kb: size of the region in kilobases.
