# Supplementary material for: Looking the Cow in the Eye: Deletion in the NID1 Gene Is Associated with Recessive Inherited Cataract in Romagnola Cattle
Source: PLoS One. 2014 Oct 27;9(10):e110628. doi: 10.1371/journal.pone.0110628 (PMC4210201; doi:10.1371/journal.pone.0110628)
Supplement: Table S3 — List of all the variants in the BTA 28 candidate region. Non-synonymous variants are bolded. (PDF) [file pone.0110628.s007.pdf]

Table S3 List of all the variants in the candidate region. Non-synonymous variants are bolded.

CHROM	POS	REF	ALT	Effect	Gene	exonRank
28	3039955	T	C	DOWNSTREAM	Q0VC87_BOVIN	
				UTR_3_PRIME	Q0VC87_BOVIN	6
28	3040356	T	C	DOWNSTREAM	Q0VC87_BOVIN	
				UTR_3_PRIME	Q0VC87_BOVIN	6
28	3040696	T	C	UTR_3_PRIME	Q0VC87_BOVIN	6
28	3188097	G	A	EXON	ENSBTAG00000012132	1
28	3333880	A	AT	UTR_3_PRIME	ENSBTAG00000010500	4
28	3390913	A	G	SYNONYMOUS_CODING	GNPAT	18
28	3390916	**C**	**T**	**NON_SYNONYMOUS_CODING**	**GNPAT**	**18**
28	3412039	**C**	**T**	**NON_SYNONYMOUS_CODING**	**GNPAT**	**6**
28	3418467	**C**	**T**	**NON_SYNONYMOUS_CODING**	**C1orf131**	**1**
				UPSTREAM	GNPAT	
28	3418502	**C**	**A**	**NON_SYNONYMOUS_CODING**	**C1orf131**	**1**
				UPSTREAM	GNPAT	
28	3418503	**A**	**G**	**NON_SYNONYMOUS_CODING**	**C1orf131**	**1**
				UPSTREAM	GNPAT	
28	3448465	T	C	SYNONYMOUS_CODING	C1orf131	5
28	3460901	GC	G	INTRON	TRIM67	14
28	3519455	T	C	SYNONYMOUS_CODING	TRIM67	2
28	3519609	**A**	**G**	**NON_SYNONYMOUS_CODING**	**TRIM67**	**2**
28	3777122	**A**	**G**	**NON_SYNONYMOUS_CODING**	**TTC13**	**15**
28	3780843	C	T	SYNONYMOUS_CODING	TTC13	17
28	3793749	T	C	UTR_3_PRIME	TTC13	23
28	3962482	T	C	INTRON	ENSBTAG00000045932	8
				INTRON	ENSBTAG00000045932	12
		**T**	**C**	**NON_SYNONYMOUS_CODING**	**ENSBTAG00000045932**	**9**
28	3962491	C	T	INTRON	ENSBTAG00000045932	8

				INTRON	ENSBTAG00000045932	12
		**C**	**T**	**NON_SYNONYMOUS_CODING**	**ENSBTAG00000045932**	**9**
28	3962516	G	T	INTRON	ENSBTAG00000045932	8
				INTRON	ENSBTAG00000045932	12
28	3962644	A	G	INTRON	ENSBTAG00000045932	8
				**NON_SYNONYMOUS_CODING**	**ENSBTAG00000045932**	**8**
				**NON_SYNONYMOUS_CODING**	**ENSBTAG00000045932**	**11**
28	3962745	C	T	INTRON	ENSBTAG00000045932	8
				**NON_SYNONYMOUS_CODING**	**ENSBTAG00000045932**	**7**
				**NON_SYNONYMOUS_CODING**	**ENSBTAG00000045932**	**10**
28	3963070	**G**	**T**	**NON_SYNONYMOUS_CODING**	**ENSBTAG00000045932**	**8**
				**NON_SYNONYMOUS_CODING**	**ENSBTAG00000045932**	**6**
28	3963654	**T**	**C**	**NON_SYNONYMOUS_CODING**	**ENSBTAG00000045932**	**6**
				**NON_SYNONYMOUS_CODING**	**ENSBTAG00000045932**	**5**
				**NON_SYNONYMOUS_CODING**	**ENSBTAG00000045932**	**6**
28	3963696	**C**	**A**	**NON_SYNONYMOUS_CODING**	**ENSBTAG00000045932**	**6**
				**NON_SYNONYMOUS_CODING**	**ENSBTAG00000045932**	**5**
28	3963759	T	C	INTRON	ENSBTAG00000045932	5
				**NON_SYNONYMOUS_CODING**	**ENSBTAG00000045932**	**5**
				SYNONYMOUS_CODING	ENSBTAG00000045932	5
28	3963829	**C**	**T**	**NON_SYNONYMOUS_CODING**	**ENSBTAG00000045932**	**5**
				**NON_SYNONYMOUS_CODING**	**ENSBTAG00000045932**	**4**
28	3963857	T	C	INTRON	ENSBTAG00000045932	4
				**NON_SYNONYMOUS_CODING**	**ENSBTAG00000045932**	**4**
				**SPLICE_SITE_ACCEPTOR**	**ENSBTAG00000045932**	**5**
28	3963871	T	C	INTRON	ENSBTAG00000045932	4
				**NON_SYNONYMOUS_CODING**	**ENSBTAG00000045932**	**4**
28	3964067	G	GA	INTRON	ENSBTAG00000045932	3
				**SPLICE_SITE_ACCEPTOR**	**ENSBTAG00000045932**	**4**
				**SPLICE_SITE_DONOR**	**ENSBTAG00000045932**	**3**

28	3964083	T	C	INTRON	ENSBTAG00000045932	3
				**NON_SYNONYMOUS_CODING**	**ENSBTAG00000045932**	**3**
28	3964152	**G**	**A**	**NON_SYNONYMOUS_CODING**	**ENSBTAG00000045932**	**3**
28	3964205	C	T	SYNONYMOUS_CODING	ENSBTAG00000045932	3
28	3964227	**C**	**T**	**NON_SYNONYMOUS_CODING**	**ENSBTAG00000045932**	**3**
28	3964244	G	A	SYNONYMOUS_CODING	ENSBTAG00000045932	3
28	3964276	**T**	**A,G**	**STOP_GAINED**	**ENSBTAG00000045932**	**3**
				SYNONYMOUS_CODING	ENSBTAG00000045932	3
28	3964290	**T**	**C**	**NON_SYNONYMOUS_CODING**	**ENSBTAG00000045932**	**3**
28	3964321	**C**	**T**	**NON_SYNONYMOUS_CODING**	**ENSBTAG00000045932**	**3**
28	3964341	T	C	INTRON	ENSBTAG00000045932	2
				**SPLICE_SITE_ACCEPTOR**	**ENSBTAG00000045932**	**3**
28	3964342	G	A	INTRON	ENSBTAG00000045932	2
28	3964374	A	G	INTRON	ENSBTAG00000045932	2
				**NON_SYNONYMOUS_CODING**	**ENSBTAG00000045932**	**2**
28	3964425	G	A	INTRON	ENSBTAG00000045932	1
				**NON_SYNONYMOUS_CODING**	**ENSBTAG00000045932**	**2**
28	3964557	G	A	SYNONYMOUS_CODING	ENSBTAG00000045932	1
28	3964585	**A**	**G**	**NON_SYNONYMOUS_CODING**	**ENSBTAG00000045932**	**1**
28	3987172	**T**	**C**	**NON_SYNONYMOUS_CODING**	**ENSBTAG00000039845**	**5**
28	3987441	G	A	SYNONYMOUS_CODING	ENSBTAG00000039845	5
28	3987483	**C**	**G**	**NON_SYNONYMOUS_CODING**	**ENSBTAG00000039845**	**5**
28	3987566	A	G	SYNONYMOUS_CODING	ENSBTAG00000039845	4
28	3987683	C	T	INTRON	ENSBTAG00000039845	3
28	3987860	**C**	**G**	**NON_SYNONYMOUS_CODING**	**ENSBTAG00000039845**	**2**
28	4122112	**G**	**A**	**NON_SYNONYMOUS_CODING**	**ENSBTAG00000025021**	**1**
28	4262866	C	G	INTRON	ENSBTAG00000025021	6
28	4263657	T	C	SYNONYMOUS_CODING	ENSBTAG00000025021	7
28	4263753	G	C	SYNONYMOUS_CODING	ENSBTAG00000025021	7
28	5292858	**A**	**G**	**NON_SYNONYMOUS_CODING**	**SIPA1L2**	**13**

28	5292897	**C**	**T**	**NON_SYNONYMOUS_CODING**	**SIPA1L2**	**13**
28	5293106	C	T	SYNONYMOUS_CODING	SIPA1L2	13
28	5299373	C	T	SYNONYMOUS_CODING	SIPA1L2	9
28	5347581	**A**	**G**	**NON_SYNONYMOUS_CODING**	**SIPA1L2**	**2**
28	5713156	C	T	SYNONYMOUS_CODING	K1383_BOVIN	1
28	5872194	T	C	UTR_5_PRIME	F1MM52_BOVIN	1
28	5872208	G	T	UTR_5_PRIME	F1MM52_BOVIN	1
28	5872247	T	C	UTR_5_PRIME	F1MM52_BOVIN	1
28	5872313	G	T	UTR_5_PRIME	F1MM52_BOVIN	1
28	5878021	T	C	SYNONYMOUS_CODING	F1MM52_BOVIN	2
28	5909820	G	A	INTRON	F1MM52_BOVIN	4
28	5910015	C	G	UTR_3_PRIME	F1MM52_BOVIN	5
28	5910417	GTT	G	UTR_3_PRIME	F1MM52_BOVIN	5
28	5910473	G	A	DOWNSTREAM	F1MM52_BOVIN	
				INTERGENIC		
28	5917639	A	G	SYNONYMOUS_CODING	PCNXL2	33
28	5984896	A	G	SYNONYMOUS_CODING	PCNXL2	25
28	6054537	G	A	SYNONYMOUS_CODING	PCNXL2	21
28	6083098	**C**	**T**	**NON_SYNONYMOUS_CODING**	**PCNXL2**	**19**
28	6084058	**A**	**G**	**NON_SYNONYMOUS_CODING**	**PCNXL2**	**18**
28	6168134	A	G	SYNONYMOUS_CODING	PCNXL2	9
28	6243190	**A**	**G**	**NON_SYNONYMOUS_CODING**	**ENSBTAG00000021183**	**1**
28	6298743	C	T	SYNONYMOUS_CODING	ENSBTAG00000021183	13
28	6559804	T	C	UTR_3_PRIME	KCNK1_BOVIN	3
28	7185945	T	C	SYNONYMOUS_CODING	SLC35F3	5
28	7188541	T	C	INTRON	SLC35F3	5
28	7188752	G	A	INTRON	SLC35F3	6
28	7195511	C	T	SYNONYMOUS_CODING	SLC35F3	8
28	7195646	T	C	SYNONYMOUS_CODING	SLC35F3	8
28	7282915	A	G	SYNONYMOUS_CODING	TARBP1	10

28	7292308	C	A	INTRON	TARBP1	7
28	7296396	G	A	SYNONYMOUS_CODING	TARBP1	6
28	7304942	C	T	SYNONYMOUS_CODING	TARBP1	2
28	7304960	T	C	SYNONYMOUS_CODING	TARBP1	2
28	7304965	**C**	**T**	**NON_SYNONYMOUS_CODING**	**TARBP1**	**2**
28	7307397	G	A	SYNONYMOUS_CODING	TARBP1	1
28	7446928	**T**	**G**	**NON_SYNONYMOUS_CODING**	**IRF2BP2**	**1**
28	7928014	G	A	UTR_3_PRIME	TOM20_BOVIN	5
28	7928096	G	T	UTR_3_PRIME	TOM20_BOVIN	5
28	7946879	G	GA	UTR_3_PRIME	A8NJ74_BOVIN	12
28	8022387	G	C	INTRON	ARID4B	9
28	8022387	**G**	**C**	**SPLICE_SITE_ACCEPTOR**	**ARID4B**	**10**
28	8046931	T	TA	INTRON	ARID4B	3
28	8210510	G	A	SYNONYMOUS_CODING	TBCE_BOVIN	4
28	**8223073**	**C**	**T**	**NON_SYNONYMOUS_CODING**	**TBCE_BOVIN**	**7**
28	8232316	G	A	INTRON	TBCE_BOVIN	15
28	8232316	G	A	UTR_3_PRIME	A7YY59_BOVIN	13
28	8342799	T	C	UTR_3_PRIME	GNG4	4
28	8424006	A	G	UTR_3_PRIME	LYST	51
28	8424588	TA	T	INTRON	LYST	50
28	8450923	G	A	SYNONYMOUS_CODING	LYST	42
28	**8492389**	**G**	**A**	**NON_SYNONYMOUS_CODING**	**LYST**	**26**
28	8508758	GAAA	GA,G, GAA	INTRON	LYST	18
28	**8664873**	**A**	**G**	**EXON**	**U6**	**1**
28	8733389	A	ATG	UTR_3_PRIME	NID1	31
28	8733559	G	A	SYNONYMOUS_CODING	NID1	31
28	8733580	G	A	SYNONYMOUS_CODING	NID1	31
28	8796044	A	C	SYNONYMOUS_CODING	NID1	7
28	8798180	**T**	**C**	**NON_SYNONYMOUS_CODING**	**NID1**	**3**
28	8798200	**A**	**G**	**NON_SYNONYMOUS_CODING**	**NID1**	**3**

28	8798242	**A**	**G**	**NON_SYNONYMOUS_CODING**	**NID1**	**3**
28	8798266	**G**	**A**	**NON_SYNONYMOUS_CODING**	**NID1**	**3**
28	8917765	A	G	SYNONYMOUS_CODING	GPR137B	3
28	8942111	A	G	DOWNSTREAM	GPR137B	
				INTERGENIC		
28	9026323	**A**	**C**	**NON_SYNONYMOUS_CODING**	**ERO1LB**	**1**
28	9242413	A	G	DOWNSTREAM	Q2YDD7_BOVIN	
				UTR_3_PRIME	Q2KIF9_BOVIN	44
28	9250434	**G**	**C**	**NON_SYNONYMOUS_CODING**	**Q2KIF9_BOVIN**	**34**
28	9264455	A	G	SYNONYMOUS_CODING	Q2KIF9_BOVIN	20
28	9269959	**G**	**A**	**NON_SYNONYMOUS_CODING**	**Q2KIF9_BOVIN**	**17**
28	9273258	T	C	SYNONYMOUS_CODING	Q2KIF9_BOVIN	14
28	9273312	T	A	INTRON	Q2KIF9_BOVIN	13
28	9441112	T	C	SYNONYMOUS_CODING	ACTN2	15
28	9483580	**A**	**G**	**NON_SYNONYMOUS_CODING**	**MTR**	**2**
28	9483679	**G**	**A**	**NON_SYNONYMOUS_CODING**	**MTR**	**2**
